# Calcium Phosphate Ceramics Can Prevent Bisphosphonate-Related Osteonecrosis of the Jaw

**DOI:** 10.3390/ma13081955

**Published:** 2020-04-22

**Authors:** Siri Paulo, Mafalda Laranjo, Anabela Paula, Ana Margarida Abrantes, João Martins, Carlos Miguel Marto, Ana Coelho, João Casalta-Lopes, Lina Carvalho, Eunice Carrilho, Arménio Serra, Maria Filomena Botelho, Manuel Marques Ferreira

**Affiliations:** 1 Institute of Endodontics, Faculty of Medicine, University of Coimbra, 3000-075 Coimbra, Portugal; 2 Coimbra Institute for Clinical and Biomedical Research (iCBR) Area of Environment, Genetics and Oncobiology (CIMAGO), Faculty of Medicine, University of Coimbra, 3000-548 Coimbra, Portugal; mafaldalaranjo@gmail.com (M.L.); anabelabppaula@sapo.pt (A.P.); amabrantes@fmed.uc.pt (A.M.A.); cmiguel.marto@uc.pt (C.M.M.); anasofiacoelho@gmail.com (A.C.); lcarvalho@huc.min-saude.pt (L.C.); eunicecarrilho@gmail.com (E.C.); mfbotelho@fmed.uc.pt (M.F.B.); 3 Center for Innovative Biomedicine and Biotechnology (CIBB), University of Coimbra, 3000-548 Coimbra, Portugal; 4 Clinical and Academic Centre of Coimbra (CACC), 3004-561 Coimbra, Portugal; 5 Institute of Biophysics, Faculty of Medicine, University of Coimbra, 3000-548 Coimbra, Portugal; 6 Institute of Integrated Clinical Practice, Faculty of Medicine, University of Coimbra, 3000-075 Coimbra, Portugal; 7 Department of Endodontology, Academic Center of Dentistry Amsterdam, University of VU Amsterdam, De Boelelaan 1105, 1081 HV Amsterdam, The Netherlands; jfbrochado@gmail.com; 8 Institute of Experimental Pathology, Faculty of Medicine, University of Coimbra, 3000-548 Coimbra, Portugal; 9 Radiation Oncology Service, Coimbra Hospital and University Centre, 3004-561 Coimbra, Portugal; joao.casalta@gmail.com; 10 Institute of Anatomical and Molecular Pathology, Faculty of Medicine, University of Coimbra, 3004-504 Coimbra, Portugal; 11 Department of Chemical Engineering, Faculty of Sciences and Technology, University of Coimbra (FCTUC), 3030-790 Coimbra, Portugal; armenio.serra@gmail.com

**Keywords:** animal models, bisphosphonate-related osteonecrosis of the jaw (BRONJ), biphasic calcium phosphates (BCP), zoledronate

## Abstract

Bisphosphonate-associated osteonecrosis of the jaw (BRONJ), a post-surgical non-healing wound condition, is one of the most common side effects in patients treated with nitrogen-containing bisphosphonates. Its physiopathology has been related with suppression of bone turnover, of soft tissue healing and infection. Biphasic calcium phosphates (BCP) are used as a drug delivery vehicle and as a bone substitute in surgical wounds. Due to their capacity to adsorb zoledronate, it was hypothesized these compounds might have a protective effect on the soft tissues in BRONJ wounds. To address this hypothesis, a reproducible in vivo model of BRONJ in Wistar rats was used. This model directly relates chronic bisphosphonate administration with the development of osteonecrosis of the jaw after tooth extraction. BCP granules were placed in the alveolus immediately after tooth extraction in the test group. The animals were evaluated through nuclear medicine, radiology, macroscopic observation, and histologic analysis. Encouragingly, calcium phosphate ceramics were able to limit zoledronate toxicity in vivo and to favor healing, which was evidenced by medical imaging (nuclear medicine and radiology), macroscopically, and through histology. The studied therapeutic option presented itself as a potential solution to prevent the development of maxillary osteonecrosis.

## 1. Introduction

The survival of cancer patients increased substantially due to the availability and efficiency of treatments. However, certain therapies determine co-morbidities and side effects, as is the case of the osteonecrosis of the jaw. Bisphosphonate-related osteonecrosis of the jaw (BRONJ) is a post-surgical lesion, in which bone exposure in the maxillofacial region persists for more than 8 weeks, in patients undergoing or underwent bisphosphonate treatment and without history of head and neck radiation [1,2]. The pathophysiology of BRONJ is related to the suppression of bone remodeling induced by bisphosphonates, the bisphosphonate toxicity itself in the surrounding tissues, and eventual local infection, which altogether maintains and perpetuates bone exposure following oral surgery involving bone tissue [3].

Although the lack of epidemiological studies, BRONJ incidence is reported from 0.8–12%, being more frequent in patients with bone metastasis who underwent intravenous nitrogenous bisphosphonate therapy [4,5]. The incidence peak in these patients is explained because bisphosphonates are effective drugs, widespread in the treatment of hypercalcemia secondary to malignancy, skeletal-related events connected with bone metastases, lytic lesion related to multiple myeloma, osteoporosis, osteopenia, and Paget’s disease, among others [6,7]. BRONJ development occurs mostly due to iatrogenic interventions, mainly tooth extractions, but also apical and periodontal surgeries and implant placement. Other factors—such as the intravenous administration of the drugs, infection, and genetic predisposition—can also promote the disease occurrence [8].

BRONJ is a highly debilitating disease, implicating prolonged pain, tooth loss, infection, fistula, difficulties with speech and mastication, and even pathologic jaw fracture [8]. Because of this, a severe impact on patient quality of life and psychological welfare exists. Currently, there are no effective strategies to reverse this condition. Conservative treatment is the core approach and may alleviate symptoms for some time. This includes pain control and optimized hygiene, mouthwashes, and antibiotics. Surgical treatment of permanent bone defects might be highly mutilating. In fact, prevention of BRONJ triggering events is the best practice. Guidelines advise against any oral surgical intervention in patients undergoing or who have undergone intravenous administration of nitrogenous bisphosphonates [1,9].

Various experimental therapies were proposed for BRONJ treatment, aiming to promote gingival wound healing, as local application of adipose-derived stem cells, of growth factors, of platelet rich fibrine, or low-level laser therapy [10,11,12]. Improved extraction socket healing was achieved by local application of a rescue bisphosphonates treatment able to decrease its bioavailability in the jawbone surfaces [13]. The background of these therapies is the assumption that bisphosphonates impair keratinocyte and fibroblast function and reduces soft tissue capacity of healing and vascularization.

Based on this information, we showed in a previous study that human gingival fibroblasts were sensitive to a bisphosphonate drug, Zoledronate (ZOL) and that biphasic calcium phosphate ceramics consisting of 75% hydroxyapatite and 25% β-TCP (BCP) reduced or abolished this toxicity, showing a significant protective effect regarding metabolic activity, cell viability, and cell migration [14]. Once calcium phosphate compounds have the ability to adsorb bisphosphonates, namely when used as bisphosphonate carriers, and are liable to be applied in surgical sites, consisting of suitable bone substitutes, we hypothesized that the application of these ceramics in a surgical site could have a protective effect from BRONJ development. Thus, the objective of this work was to evaluate an animal model of BRONJ through imaging and to evaluate the protective potential of BCP on BRONJ development.

## 2. Materials and Methods

### 2.1. Ethical Statement

This study was approved by the Ethics Committee of the Faculty of Medicine of the University of Coimbra (ref. 24-CE-2011) and performed according to the European Guidelines and Portuguese Law. Animals were cared according to the recommendations presented in the Guide for the Care and Use of Laboratory Animals of the National Institutes of Health. The experimental protocols were performed and reported according to the ARRIVE guidelines [15,16].

### 2.2. Experimental Animals

Thirty-five female Wistar rats, aged 16–18 weeks with an average weight of 248.74 ± 28.52 g, were provided by the animal facility of the Faculty of Medicine, University of Coimbra. Housing was carried in individually ventilated cages, under a 12-h light/dark cycle, with access to standard diet and filtered water ad libitum. Paper rolls and strips were provided as environmental enrichment. Animals were distributed in a randomized manner through the study groups described below and identified by earmarking. Three times per week, animal welfare was checked, and no significant issues were found throughout the study timeline.

### 2.3. Study Design

The animals were split into five groups, as shown in Table 1. The BRONJ development was performed considering previous reports using nitrogenous bisphosphonates [17,18,19]. Thus, the animals were administered intraperitoneally with an aqueous solution of zoledronate, 0.1 mg/kg, three times a week, until the end of the study (maximum of 7 weeks). In the fourth week, the first lower left molar was extracted. Saline solution was administered instead of zoledronate at the control groups. The treatment group received a BCP local application at the surgical wound, immediately after tooth extraction. The BCP used was Adbone^®^BCP, (Medbone^®^ Medical Devices, Sintra, Portugal), a porous synthetic bone biomaterial, constituted by 75% hydroxyapatite (HA) and 25% beta phosphate tricalcium (β-TCP), having a porosity of 80% with a pore size in the range of 300–500 microns.

Part of the animals were killed two weeks after tooth extraction: seven animal models of BRONJ (ZOL), and seven animals as control. The rest of the animals were killed three weeks after tooth extraction: seven animal models of BRONJ (ZOL), seven animal models of BRONJ with BCP application (ZOL/BCP), and seven animals as control.

### 2.4. Surgical Procedure

The surgical procedure was performed under deep anesthesia of the animals through an intraperitoneal injection of 2 μL g of a 3:1 solution of ketamine (50 mg/mL; Ketalar^®^, Parke-Davis, Pfizer Laboratories Lda. (New York, NY, USA) and chlorpromazine (2.5%, Largactil^®^, Rhône-Poulenc Rorer, Paris, France).

**Figure 1:**
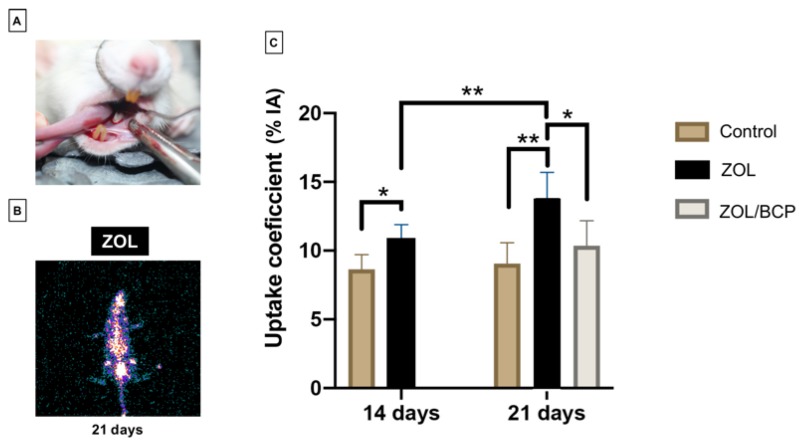
Animal model and results of nuclear medicine: (**A**) surgical intervention; (**B**) illustrative figure of the functional imaging with ^99m^Tc-ZOL of an animal of ZOL group acquired with a gamma-camera; (**C**) Uptake coefficients, calculated as the ratio between the maximum counts of the left mandible (with the surgery) and the average counts of the right mandible (control). Results are shown as the mean ± standard deviation. Significant differences are marked with * *p* < 0.05, ** *p* < 0.01, *** *p* < 0.001.

The animals were placed in the prone position, keeping the mouth open with the support of a metal ring anchored on the upper incisors, so that the upper jaw was stretched and immobilized (Figure 1A). The device also provided support of the lower jaw, with cheek dislocation, with a retractor adapted for this purpose, keeping away the tongue with hemostatic forceps. A surgical magnifying glass was used in all interventions, in order to allow adequate visualization of the operative field.

To perform the tooth extraction, a periodontal curette was adapted. Gentle movements were applied in the tooth, and traction was performed with the aid of a hemostatic forceps with an active part appropriate to the size of the animals’ teeth. At the end of the surgical procedure, the wound was sutured with a 4/0 resorbable thread (Surgilactin, Sutura Lda, Wales, UK), assisted by a needle holder and surgical scissors. In the group in which BCP (Adbone^®^BCP, Medbone^®^ Medical Devices, Sintra, Portugal) was applied, the procedure was identical to the described, with granules of BCP being placed in the alveolus, with subsequent suture.

### 2.5. Nuclear Medicine Imaging

Functional imaging was obtained using ^99m^Tc-zoledronate (^99m^Tc-ZOL) prior animal occision. The labeling procedure was performed according to Asikoglu et al. [20]. For the preparation of the radiopharmaceutical (^99m^Tc-ZOL), a solution with the following components was prepared: 1mL of zoledronate 5 mg/mL, 0.4 mL of ascorbic acid 0.01 g/mL, 0.5 mL of dehydrated stannous chloride 1mg/mL. For this, ultra-pure water was used, and the final solution was degassed with argon. For labeling, sodium pertechnetate activity (130–160 MBq) was added. Labeling quality control was performed using two thin layer micro chromatography systems: W3MM paper (Whatman) as a stationary phase and acetone or saline as mobile phases. ^99m^Tc-ZOL with a labeling efficiency higher than 90% was used for imaging.

For image acquisition, the animals were anesthetized with the drugs referred above. Administration was performed in the tail vein and 90 min later, images were acquired with a gamma camera (GE, Milwaukee, WI, USA), coupled with a high-resolution, low-energy parallel collimator. Static images were collected using 256 × 256 matrix, for 2 min. Energy discrimination was centered on 140 keV, with a 20% energy window. Image processing was performed in an eNTEGRA workstation by tracing the regions of interest (ROIs) in jaws. The uptake coefficient, the ratio between the maximum counts of the left mandible (with the surgery) and the average counts of the right mandible (control), was calculated as described by Soler et al. [21]. The uptake coefficients are shown as percentage of injected activity (%IA).

### 2.6. Macroscopic Evaluation of Specimens

The macroscopic evaluation, accompanied by the photographic record, evaluated clinical signs of healing in the surgical intervention’s region; the presence or absence of a mucosal continuity solution; as well as the existence of bone exposure, ulcerations, abscesses, purulent drainage or other clinical signs. Inspection considered the mandibles, the surrounding soft tissues, and also the peri-oral region.

### 2.7. Radiographic Evaluation

After jaw dissection, radiographs of the hemi-mandibles were obtained. For this, a portable X-ray generating source Por-X II device (Genoray Co. Lda, Seongnam-Si, Gyeonggi-Do, Korea), with cone D-081B (Toshiba, Tokyo, Japan) was used. Radiographs were analyzed with ImageJ software (version 1.8.0_172, NIH, Bethesda, MD, USA). Regions of interest (ROI) were drawn and, to obtain the attenuation coefficient, the ratio between the average values in the left mandible (with the surgery) mandible and in the right mandible (control) was calculated.

### 2.8. Histological Evaluation

After fixing the samples in a 10% buffered formaldehyde solution for 24 h, they were washed in running water and then decalcified with Morse’s solution, at a temperature of 4 °C, for about 10 days. After this period, the hemi-mandibles were sectioned in the sagittal direction. Conventional dehydration and inclusion in paraffin were carried out. The paraffin blocks were sectioned (Shandon Finesse 325^®^ microtome, Thermo Electron Corporation, Waltham, MA, USA) and slides were stained with hematoxylin-eosin (H&E), for routine histological analysis.

Histological observations were performed randomly and blindly using a bright-field optical microscope (Nikon 600 D, Nikon, Tokyo, Japan) coupled with high-resolution digital photographic equipment. The analysis was based on the evaluation of the healing process, changes in the level of the fibrin network, blood cells, vascularization, the presence or absence of inflammatory cells, granulation tissue, fibrous connective tissue, activity of the osteoblasts and osteoclasts, bone remodeling, bone trabeculae, vital or necrotic bone tissue, and the integrity of the covering epithelium.

### 2.9. Statistical Analysis

For the animal study, prior power analysis was performed considering a power of 0.8 and an effect size of 0.65, in the G*power software. The statistical analysis was performed using the IBM^®^ SPSS^®^ version 20 software (IBM, Armonk, NY, USA). Descriptive analysis was expressed using measures of central tendency and dispersion for quantitative variables. In the inferential analysis, the Shapiro–Wilk test was used for normality evaluation. For the results of the 14 days, the comparison between the control and zoledronate conditions was performed according to the Student’s *t*-test for independent samples (parametric test) or Mann–Whitney test (non-parametric test). At 21 days, the control, zoledronate, and zoledronate/BCP conditions were compared, so the one-way ANOVA test (parametric test) was used, in case of normal distribution and homogeneity of variances, or the Kruskal–Wallis test (non-parametric test) otherwise. Multiple comparisons were performed with Bonferroni correction. A significance value of 5% was considered for all comparisons.

## 3. Results

### 3.1. Nuclear Medicine

^99m^Tc-Zoledronate was used with a labeling efficiency greater than 90% (92.0 ± 1.6%). The quality control evaluation revealed that the radiopharmaceutical remains stable, with efficiency greater than 90% for a period of 6 h.

The uptake coefficients are shown in Figure 1. Fourteen days after tooth extraction, there was a significant increase in animal models of BRONJ (submitted to zoledronate therapy, ZOL) to 10.85 ± 1.44%IA regarding the animals of the control group, 8.64 ± 1.06%IA (*p* = 0.015). At 21 days after tooth extraction, the uptake coefficients of control group are 9.05 ± 1.53%IA, with an even higher increase in the BRONJ group (ZOL) with values of 13.7 ± 1.99%IA (*p* = 0.008). Encouragingly, the uptake coefficient of the group submitted to biphasic calcium phosphate ceramics application (ZOL/BCP) remained with a value of 10.36 ± 1.81%IA, similar to the control and significantly lower than the BRONJ (ZOL) animal models (*p* = 0.014).

### 3.2. Radiology

**Figure 2:**
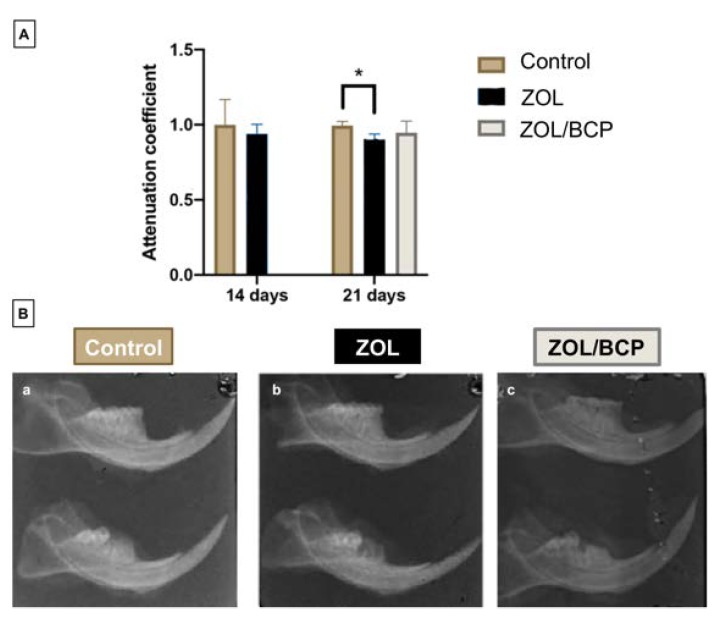
Radiographic evaluation: (**A**) Attenuation coefficients, calculated as the ratio between the average values in the left mandible (with the surgery) and in the right mandible (control). Results are showed as the mean ± standard deviation. differences are marked with * *p* < 0.05, ** *p* < 0.01, *** *p* < 0.001. (**B**) Representative radiographic images of the hemi-mandibles; on top the right mandible (control) and on the bottom the left mandible (with the surgery); (**a**) control group, (**b**) ZOL group, (**c**) ZOL/BCP group.

The radiographic attenuation coefficients are shown in Figure 2. Fourteen days after surgery there are no statistically significant differences between groups. However, 21 days after extraction, the animals in the group submitted to zoledronate therapy (ZOL, 0.902 ± 0.036) have a density coefficient significantly lower than the control group (0.996 ± 0.027; *p* = 0.001). This alteration is not observed in the group where the placement of phosphate ceramics biphasic calcium in the surgical site was performed (ZOL/BCP), which remains similar to the control.

### 3.3. Macroscopic Analysis

**Figure 3:**
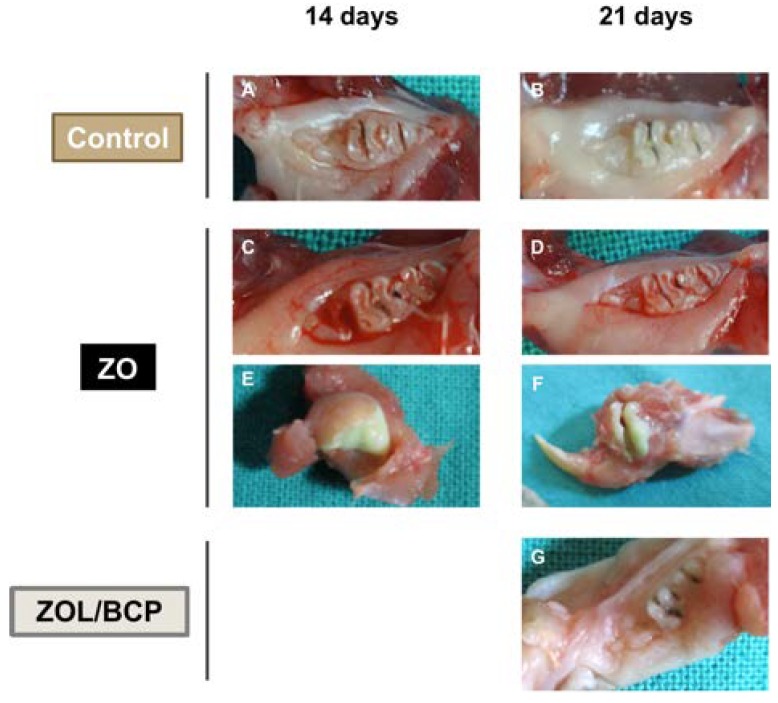
Macroscopic analysis of the mandibles. Representative images of the control group 14 (**A**) and 21 days (**B**) after tooth extraction showing the healed mucosa. Representative images of the ZOL group 14 days after tooth extraction showing a solution of continuity (**C**) and a purulent abscess (**E**), and 21 days after tooth extraction showing the incompletely healed mucosa with osseous exposition (**D**) and a purulent abscess (**F**). Representative image of the ZOL/BCP group of 21 days (**G**) after tooth extraction with healed mucosa.

Representative images of the macroscopic analysis are shown in Figure 3. The animals of both control groups killed 14 or 21 days after surgery, did not present any cases with a continuity solution. However, in animals subjected to zoledronate administration (ZOL), the presence of solution of continuity was found in 72% of the cases evaluated at the 14th day, and 57% of the cases evaluated at the 21st day. In this group of animals, the presence of abscesses with purulent content was observed in 28% of the animals (both for the 14th and 21st days). Promisingly, no continuity solutions or other clinical signs were observed in the animals where the placement of calcium phosphate bioceramics in the surgical site (ZOL/BCP) was performed.

### 3.4. Histological Analysis

**Figure 4:**
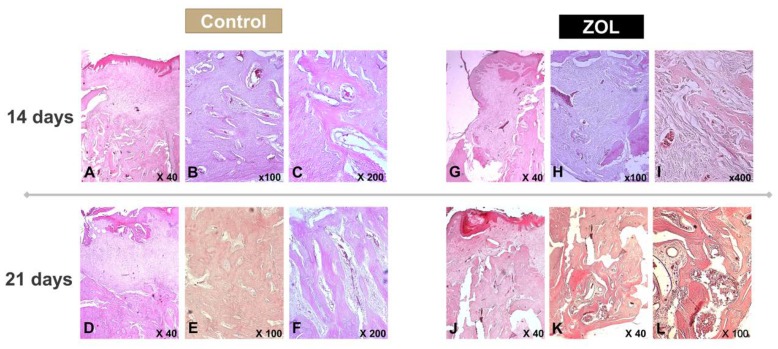
Histological images of control group 14 (**A–C**) and 21 days (**D,E**) after tooth extraction; ZOL groups 14 (**F–H**) and 21 days (**I–K**) after tooth extraction. (**A**)—complete closure of the surgical wound. Presence of stratified squamous epithelium and surgical site is filled by spongy bone tissue, H&E 40×; (**B,C**)—newly formed bone tissue in the surgical site, immature osseous tissue, and areas of lamellar osseous tissue. H&E 100x and 200×, respectively; (**D**)—complete closure of the surgical wound, with the healing of soft and hard tissues. The surgical site is filled with a large amount of bone tissue, H&E 40×; (**E**)—increase in the thickness of the bone trabeculae as well as the appearance of some Havers systems and the presence of a network of bone trabeculae, separated by a richly vascularized connective tissue, H&E 100×; (**F**)—presence of a network of bone trabeculae, separated by a richly vascularized connective tissue. The bone trabeculae are made up of areas of lamellar bone tissue covering areas of immature bone tissue, which occupy a central position, H&E 200×; (**G**)—partial covering of surgical wound with epithelial tissue and fibrous connective tissue, H&E 40×; (**H,I**)—intramembranous ossification process. It is visible a large amount of dense connective tissue occupying the extraction site and a residual proportion of osteoblasts adjacent to the bone trabeculae, H&E 100× and 400×, respectively. (**J**)—closure of the surgical wound with structural disorganization of the epithelial tissue, as well as of the newly formed bone tissue, with the presence of trabeculae of immature bone tissue with a reasonable thickness, separated by connective tissue with a certain fibrous reinforcement and some blood vessels, H&E 40×; (**K,L**)—apical region of the surgical site, where a smaller thickness of the bone trabeculae is identified, however, between areas of lamellar bone tissue. Image suggestive of osteomyelitis, H&E 40× and 100×, respectively.

Slides from the control group, occision 14 days after surgery, evidenced the normal process of soft and hard tissue healing after extraction. The soft tissue healed with a stratified keratinized squamous epithelium, with typical morphology of the oral epithelium, indicating the complete closure of the surgical wound (Figure 4A). It is also evident the presence of large areas of new bone tissue occupying a large extent of the surgical defect (Figure 4A,B). The bone tissue presents the typical morphology of immature bone tissue, with a high density of osteocytes, lodged in rounded gaps, with a disorganized disposition. The spaces between the newly formed trabeculae are occupied by loose connective tissue, with a rich vascularization (Figure 4C). Bone remodeling processes are already visible, translated by the presence of some osteoclasts and the appearance of lamellar bone tissue (Figure 4C).

In the slides from the control group, occision 21 days after surgery, complete closure of the surgical wound is observed (Figure 4D). It is possible to identify a considerable increase in bone neoformation, characterized by an increase in the thickness of the trabeculae, and remodeling processes. The resorption of immature bone trabeculae, in parallel with the formation of lamellar tissue, were evident. The newly formed bone tissue now occupies the entire area of the defect, establishing a perfect continuity with the walls of the socket and progressively approaching the normal architecture of compact bone tissue (Figure 4E,F).

The majority of samples from BRONJ group (ZOL, occision 14 days after surgery) showed the formation of a keratinized stratified squamous epithelium (Figure 4G), but the surgical site is essentially filled with fibrous connective tissue rich in cells, with no clear signs of bone formation (Figure 4H). Nevertheless, it is possible to observe some osteogenic activity adjacent to the interdental septum, characterized by thin bony trabeculae surrounded by cells with osteoblastic characteristics, but in a very residual proportion (Figure 4I). After 21 days of evolution, there is a change in the morphology of the epithelium, characterized by a decrease in its thickness as well as less interdigitation with the underlying lamina propria (Figure 4J). Foremost, in some samples, areas of discontinuity of the mucosa were evident, showing the absence of closure of the surgical wound as well as the absence of bone tissue in the extraction site.

It should also be noted that in the most coronal region of the surgical site, it is possible to observe the presence of trabeculae of immature bone tissue with a reasonable thickness, separated by connective tissue with a certain fibrous reinforcement (Figure 4J). As we move apically in the surgical site, there is a decrease in bone trabeculae thickness, but its structure is mostly of areas of lamellar bone tissue (Figure 4K). The connective tissue between the osseous trabeculae shows a number of hyperemic vessels as well as some hemorrhagic zones and some cells with inflammatory characteristics, which is suggestive of osteomyelitis (Figure 4L). In the most apical region of the defect, there are areas of necrotic bone tissue characterized by the absence of osteocytes within their lacunae. However, in some cases, the direct apposition of immature bone tissue on these necrotic bone trabeculae can be visible.

**Figure 5:**
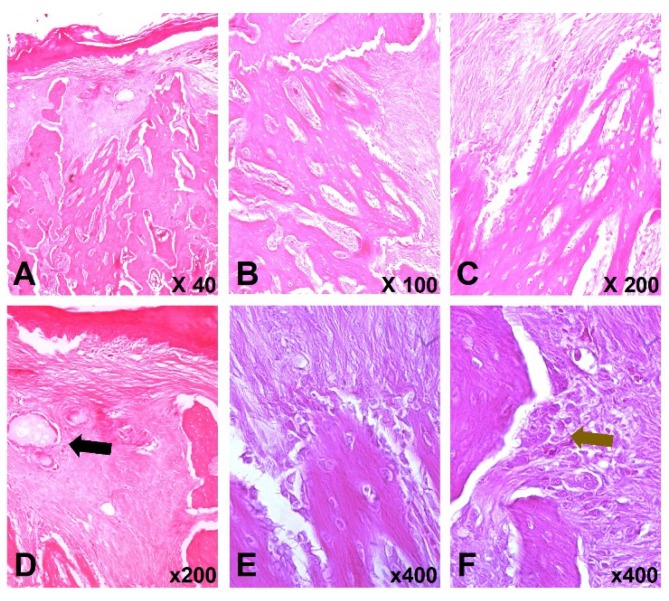
Histological images of the ZOL/BCP group 21 days after tooth extraction. (**A**)—continuity of the mucosa is observed, as well as bone neoformation at the site of extraction, H&E 40×. (**B,C**)—bone neo-formation in the surgical site, with the presence of numerous bone trabeculae occupying a large extension of the site, with progression from apical to coronal. The most coronal region is occupied by fibrovascular tissue. Presence of recently formed bone trabeculae and it is possible to observe, in addition to the osteoblastic layer, a large density of osteocytes arranged irregularly and housed in rounded gaps, a typical aspect of immature bone tissue, H&E 100× and 200×, respectively. (**D**)—fragments of the previously placed regeneration material are rarely visible—black arrow, H&E 200×. (**E**)—intramembranous ossification process, H&E 400×. (**F**)—presence of cells with an osteoclastic profile–brown arrow, showing an active process of bone resorption/remodelling, H&E 400×.

In the group where the placement of calcium phosphate bioceramics in the surgical site was performed (ZOL/BCP), it was observed complete healing of the soft tissue with its typical structure, stratified keratinized squamous epithelium (Figure 5A). Regarding the bony defect, it is worth mentioning the presence of areas of bone tissue filling, in a significant extension of the surgical defect. It was not frequently detected the presence of fragments of the regeneration material used (Figure 5A,D, black arrow). The formation of new bone tissue by intramembranous ossification is translated by the presence of a network of thin bone trabeculae, progressing from the margins of the surgical site, in the coronal direction. The most superficial region is essentially occupied by fibrovascular scar tissue (Figure 5B,C,E). In the newly formed trabecular bone is possible to observe besides the osteoblastic layer, a large density of osteocytes arranged irregularly, a typical aspect of immature bone tissue (Figure 5C). It is also to be noted, the presence of some osteoclasts reflecting the beginning of a bone remodeling process (Figure 5F, brown arrow).

## 4. Discussion

In this study, it was used a reproducible experimental model of BRONJ development in Wistar rats, which directly relates the chronic administration of bisphosphonates with the development of maxillary osteonecrosis after tooth extraction [17,18,19]. It is documented that, after tooth extraction in rats, the most intense phase of bone formation occurs after 14 days and bone resorption occurs up to 7 days [22,23,24,25,26,27,28]. Considering the definition of BRONJ as a lesion persistent for more than 8 weeks, which would correspond approximately to one week in a Wistar rat [29], the experimental protocol included two evaluation periods, thus determining the occision of one group of animals 14 days after tooth extraction and another group one week later.

After tooth extraction, the clot present in the wound space, provides a temporary matrix for cell migration where fibroblasts, epidermal cells, and endothelial cells begin to proliferate [30]. Fibroblasts initially assume a migratory phenotype, then a collagen-producing phenotype and, finally, a contractile phenotype, responsible for the scar contraction [31]. In the case of the chronic treatment with bisphosphonates, the inflammatory process leads to a decrease in pH, which favors the release of bisphosphonates from the bone reservoir to the surgical wound [32]. Toxicity to soft tissues appears to result from the bisphosphonates accumulated in the bone and released during the healing process, during osteoclast-mediated resorption, and through a decrease in pH. This delays the closure of the mucosal barrier and prolongs the deleterious effects of exposure of the underlying bone to microorganisms. Bisphosphonates are also harmful to osteoblasts, endothelial cells, and keratinocytes, which are also essential to healing.

We recently verified that human gingival fibroblasts lose their ability to migrate in the presence of zoledronate in concentrations equal or superior to 5 µM, expectable to be present in the surgical wound [14,33]. Thus, considering the effect of zoledronate on fibroblasts just after the first 24 h, this explains the early impairment of the healing process and the inability of the mucosa to cover the lesion. Results obtained at 14 and 21 days for the ZOL groups support this observation, since a delayed healing was observed.

The introduction of calcium phosphate ceramics in the surgical site was intended to promote a protective effect from zoledronate toxicity to the soft tissues. Calcium phosphate ceramics have been used as bone substitutes and as drug transport systems [34]. These materials are similar to the bone matrix and able to form chemical bonds with the living tissue. Their biocompatibility, versatility, availability, mode of biodegradation, natural bone-like composition, and cost-effectiveness are some of factors that make good bone substitutes and drug carriers [34]. In this study, we use a commercialized material, of national manufacture, with proportions of hydroxyapatite and β-TCP that present the ability to be reabsorbed and integrated by the bone. Moreover, our previous in vitro studies showed the favorable chemical interaction and ability of BCP to arrest zoledronate in the medium, protecting human fibroblast cells from the bisphosphonate toxicity [14]. Encouragingly, in this study we showed in vivo the ability of calcium phosphate ceramics to limit zoledronate toxicity favoring healing, which was evidenced by medical imaging (nuclear medicine and radiology), macroscopically and through histology.

Nuclear medicine is accurate in diagnosing maxillary bone lesions in patients with clinically established BRONJ [35,36,37,38,39]. The uptake of the radiopharmaceutical is influenced by several factors, including bone vascularization and osteogenesis. The different retention in the tissues means areas of normal uptake, overtake, or undertake [40]. This method of analysis clearly indicates, with significant statistical differences, that zoledronate delays tooth extraction wound healing, and that the presence of calcium phosphate ceramics restrains zoledronate toxicity since the nuclear medicine values in this group of animals are close to the observed in the control group.

On radiographic analysis, whether panoramic or periapical, from a certain stage of maxillary osteonecrosis, less radiopaque areas can be observed, with some radiopaque sequestration of necrotic bone tissue and little evidence of healing [41]. The radiographic image of asymptomatic patients shows a radiopaque lesion corresponding to an empty socket [42]. However, a change of 5% in bone turnover can be detected by nuclear medicine, whereas, in radiography and computed tomography, a mineral loss of 40–50% is necessary to detect a decrease in radiopacity [43]. This way, the functional bone image can detect alterations before anatomical changes are evident, with sensitivity to the initial osteolytic processes. Thus, a small amount of bone destruction allows early detection, supporting the valuable role of the performed nuclear medicine analysis [44]. Dore et al., in a study with patients, found that orthopantomography failed to detect the difference between necrotic bone and normal bone, failing to identify lesions of maxillary osteonecrosis early [45].

In any case, evaluation through radiology is one of the first diagnostic tools. Orthopantomography allows a general assessment of the jaws. However, as said, only mineral losses in the order of 40% or more can be detected. This might be the reason why no alterations are seen between BRONJ and control groups killed 14 days after surgery. Nevertheless, in the groups of animals killed 21 days after tooth extraction, a slight reduction of the attenuation coefficient is seen in the BRONJ group versus the control. The group where the introduction of calcium phosphate ceramics in the surgical site was performed presents an attenuation coefficient value similar to the corresponding control group.

Histological analysis is the gold standard for tissue examination, either for research or diagnostic purposes, and it was used to assess the inflammation and healing stages. Histology unequivocally attests the animal model of BRONJ used is effective in recapitulating the disease. Animals from the BRONJ group, killed 14 days after tooth extraction, presented incomplete soft tissue coverage of the surgical site with consequent hard tissue exposure, as well as a smaller amount of newly formed bone tissue than control samples. Additionally, in most cases of this experimental group (BRONJ, 14 days), the surgical site was filled with fibro-vascular tissue, with few signs of bone neoformation, contrary to what happened in the animals of the correspondent control group in which, despite immature bone, numerous trabeculae with a reasonable thickness were present. In a later phase of healing, considering the animals killed 21 days after tooth extraction, control samples presented structures similar to Havers’ systems, indicating the transition to a stage of mature bone tissue. On the contrary, in the samples from the experimental group of BRONJ (ZOL) mainly immature bone tissue was seen, some foci of osteomyelitis are visible, with structural and morphological disorganization of the newly formed bone tissue. This fact can be explained by the zoledronate effects on the cells involved in healing in the surgical site, as stated before. Additionally, we previously demonstrated the stable bound between BCP and Zoledronate along 120 h in vitro [14]. This time is sufficient for clot formation and for first steps of wound healing. With this study, we showed this bond to be sufficient to allow tissue healing in vivo.

In the experimental group where biphasic calcium phosphate ceramics were placed (ZOL/BCP) in the surgical wound, 100% of the animals presented a completely healed mucosa, and bone tissue had an organization practically identical to that observed in the corresponding control group, therefore presenting more mature tissues than in the BRONJ group. BCP promoted the formation of new bone tissue that progressed from the margins of the surgical site, from apical to coronal, and the presence of fibrovascular scar tissue in the more coronal region, proving the beneficial effect of calcium phosphate bioceramics. This fact was corroborated by the previously explained histological findings, and these results are in line with the results of the tests carried out in the in vitro study, in which there was a protective effect of calcium phosphate bioceramics [14].

## 5. Conclusions

The experimental model used of the present study supports that experimental groups with zoledronate administration, in which extractions were performed, demonstrate an inability of soft tissues coverage, accompanied by underlying bone exposure, suggesting that the soft tissue toxicity may contribute to the establishment of maxillary osteonecrosis. However, using biphasic calcium phosphate ceramics, it is possible to minimize the consequences of the release of bisphosphonates from the bone reservoir after extraction, favoring wound healing.

The studied therapeutic option presented itself as a potential solution to prevent the development of maxillary osteonecrosis. When arresting the zoledronate released by the bone, the biphasic calcium phosphate ceramics decreased its bioavailability. This material, because it is already known, studied, and used in dentistry, allows its potential application in the surgical site after tooth extractions in patients with bisphosphonate therapy. Thus, in the future, the use of BCP in tooth extraction sites in patients undergoing zoledronate therapy may be an option and the subject of a clinical study.

## Figures and Tables

**Table 1:** Synthesis of the experimental groups and procedures

Group		Administration	Tooth Extraction	BCP	Occision ^1^
1	Control 14	Saline	Yes	No	14 days
2	ZOL 14	Zoledronate 0.1mg/kg	Yes	No	14 days
3	Control 21	Saline	Yes	No	21 days
4	ZOL 21	Zoledronate 0.1mg/kg	Yes	No	21 days
5	ZOL/BCP	Zoledronate 0.1mg/kg	Yes	Yes	21 days

^1^ Occision was performed 14 or 21 days after tooth extraction.
